# Evaluating Global Health Partnerships: A Case Study of a Gavi HPV Vaccine Application Process in Uganda

**DOI:** 10.15171/ijhpm.2016.137

**Published:** 2016-10-26

**Authors:** Carol Kamya, Jessica Shearer, Gilbert Asiimwe, Emily Carnahan, Nicole Salisbury, Peter Waiswa, Jennifer Brinkerhoff, Dai Hozumi

**Affiliations:** ^1^Infectious Diseases Research Collaboration (IDRC), Kampala, Uganda.; ^2^PATH Seattle, WA, USA.; ^3^Makerere University School of Public Health, Kampala, Uganda.; ^4^Department of Public Health Sciences, Karolinska Institutet, Stockholm, Sweden.; ^5^The INDEPTH Network, Maternal, Newborn and Child Health Working Group, Accra, Ghana.; ^6^George Washington University, Washington, DC, USA.; ^7^Management Sciences for Health, Arlington, VA, USA.

**Keywords:** Immunization, Social Network Analysis (SNA), Partnership, Global Health, Uganda, Gavi

## Abstract

**Background:** Global health partnerships have grown rapidly in number and scope, yet there has been less emphasis on their evaluation. Gavi, the Vaccine Alliance, is one such public-private partnership; in Gavi-eligible countries partnerships are dynamic networks of immunization actors who work together to support all stages and aspects of Gavi support. This paper describes a conceptual framework – the partnership framework – and analytic approach for evaluating the perceptions of partnerships’ added value as well as the results from an application to one case in Uganda.

**Methods:** We used a mixed-methods case study design embedded in the Gavi Full Country Evaluations (FCE) to test the partnership framework on Uganda’s human papillomavirus (HPV) vaccine application partnership. Data from document review, interviews, and social network surveys enabled the testing of the relationships between partnership framework domains (context, structure, practices, performance, and outcomes). Topic guides were based on the framework domains and network surveys identified working together relationships, professional trust, and perceptions of the effectiveness, efficiency, and legitimacy of the partnership’s role in this process.

**Results:** Data from seven in-depth interviews, 11 network surveys and document review were analyzed according to the partnership framework, confirming relationships between the framework domains. Trust was an important contributor to the perceived effectiveness of the process. The network was structured around the EPI program, who was considered the leader of this process. While the structure and composition of the network was largely viewed as supporting an effective and legitimate process, the absence of the Ministry of Education (MoE) may have had downstream consequences if this study’s results had not been shared with the Ministry of Health (MoH) and acted upon. The partnership was not perceived to have increased the efficiency of the process, perhaps as a result of unclear or absent guidelines around roles and responsibilities.

**Conclusion:** The health and functioning of global health partnerships can be evaluated using the framework and approach presented here. Network theory and methods added value to the conceptual and analytic processes and we recommend applying this approach to other global health partnerships to ensure that they are meeting the complex challenges they were designed to address.

## Background

As the size and scope of the global health agenda has grown in the past decades, so too has the need to leverage a greater number and type of actors and their resources, leading to the rise of “Global Health Public-Private Partnerships,” (GHPPPs) or “Global Health Initiatives.” The World Health Organization (WHO) Maximizing Positive Synergies Collaborative Group counted 100 such entities that participate in a range of activities related to global health decision-making, funding, implementation and technical assistance at global, national, and sub-national levels.^1^ United Nations (UN) agencies and other international organizations have signaled their support for GHPPPs, perhaps, as suggested elsewhere, to fill perceived gaps in their own mandate and expertise.^2^ “Partnership” is a Sustainable Development Goal, indicating global recognition for the partnership approach to development.^3^

A review of GHPPPs suggests they are particularly effective at getting specific health issues onto national and international agendas, mobilizing additional funds for these issues, stimulating research and development, improving access to cost-effective health interventions, strengthening national policy processes and content, augmenting health service delivery capacity, and establishing international norms and standards.^4^ This list of strengths simultaneously signals the vertical, disease-specific nature of GHPPPs – a commonly reported flaw.^1^ Other observed challenges with this model include their propensity to skew national priorities, poor transparency and accountability standards, insufficient use and harmonization with country systems, leading to waste, and sub-standard approaches to managing human resources within the partnership.^4^

Gavi, the Vaccine Alliance, formerly known as the Global Alliance for Vaccines and Immunizations is an example of an early adopter of the global health partnership governance and business model. Founded in 1999 as a public-private partnership, the Alliance brings together country governments, UN agencies, vaccine manufacturers, philanthropic foundations, non-governmental organizations (NGOs) and research institutes to achieve the Alliance’s mission of saving lives and protecting people’s health by increasing access to immunization in poor countries. Each partners’ activities are mutually agreed upon at the global level and outlined in an annual business plan,^5^ with the Gavi Secretariat serving as a “steward for the Alliance” and the Gavi Alliance Board ultimately endorsing all decisions.^6^ While the Alliance has been credited with accelerating the adoption and increasing the coverage of new and under-utilized vaccines,^7,8^ its partnership approach has not been without growing pains and critiques. The first independent evaluation of the Alliance highlighted the issue of unclear roles and responsibilities but contrasted this with the partnership’s ability to “allow quick decision-making, innovation and flexibility, and open debate and self-assessment.”^9^ The second Gavi evaluation reported the benefits of a lean organizational structure on efficiency and effectiveness.^10^ Yet, Naimoli^6^ reported that in the case of gavi’s health systems strengthening (HSS) grants, inadequate explication of roles and responsibilities, a lack of transparency and trust, and differing ideologies were some challenges noted by participants at all levels of the Alliance.

As the Gavi strategy has evolved to include strategic goals related to coverage and equity as opposed to simply new vaccine introductions,^9,10^ so too has the recognition that partners must bring a growing diversity of skills and approaches to the partnership.^11,12^ This is certainly true for the case of Uganda, whose immunization system performance is gradually improving following the re-introduction of Gavi support in 2012 and thus the reorganization of its immunization program and partnership, and where the efficient allocation and coordination of partners’ comparative advantages will be essential to supporting national and global goals.^13^ Gavi’s 2015 announcement of a new structure for coordinating and managing Gavi Alliance partners — the Partners’ Engagement Framework^14^— will replace the Gavi Business Plan in an effort to ensure country-centric processes and increase the relevance of partners’ activities; it is also a reflection of the broader growth in the diversity and decentralization of partnerships and networks for global health.^5,14^ This paper presents a case study of an Alliance partnership in Uganda prior to the introduction of the Partners’ Engagement Framework, but which may serve as a useful baseline.

This paper outlines an approach to evaluating the effectiveness, efficiency, and legitimacy of global health partnerships with an illustration from the Full Country Evaluations (FCE) commissioned by the Alliance. The FCE is a 4-year prospective evaluation of Alliance support in four countries which ultimately aims to connect inputs, outputs, outcomes and the impact of Gavi support while learning lessons about the process and areas for improvement.^15^ A key evaluation question is centered on the role and effect of the Alliance partnership on decision-making, planning, and implementation of financial and technical support. This study builds on existing evaluations of the Alliance partnership^1,4,6,9,10^ by introducing a framework that illustrates the theoretical linkages from inputs to outcomes — effectiveness, efficiency, and country ownership — of partnership-led processes through the application of mixed-methods data collection and analysis. The results of this study will be of particular salience as the Partners’ Engagement Framework is implemented and may serve as a baseline description of the state of Gavi partnership in one priority country.

The aims of this paper are two-fold. First, we describe in detail how to design and implement an evaluation of health partnerships, drawing on a specific theoretical framework (the ‘partnership framework’) and social network analysis (SNA). Second, we apply the partnership framework to a specific national level case of an immunization partnership to demonstrate how the framework can be used to describe and relate partnership domains to perceptions of outcomes in the process, and to assess the feasibility and usefulness of this approach. The case study is the process surrounding the Government of Uganda’s application for Gavi funding for national introduction of the vaccine against human papillomavirus (HPV). The paper closes with a discussion of the implications of the partnership evaluation approach for other global health partnerships and partnerships more generally.

### Appropriately Defining and Measuring Partnership Practice and Structure

A broad range of literature addresses partnership definition, practice, and measurement, including concepts from public administration, organizational science, and network analysis.^16-22^ The FCE evaluation team integrated these concepts to develop and apply a new conceptual framework to measure the partnership approach’s contribution to national immunization processes. In doing so, the evaluation responds to two gaps in current partnership evaluation practice: the tendency to ignore the inner workings of partnership relationships, and limited use of available analytic tools to measure and describe partnership structure. Partnerships are composed of individuals and the relationships between them, making it essential to understand the inner workings of a partnership to influence the overall functioning of the partnership. Despite the availability of SNA tools and methods, the increasing number of global health partnerships has not been matched by a corresponding increase in measurement of these networks. This paper provides a framework whereby stakeholders or decision-makers can evaluate their own partnerships to ensure that there is an added value of the existing partnership in terms of efficiency, effectiveness, and country ownership.

### A Partnership Framework for Looking Inside the Black Box

We defined partnership according to existing definitions from public administration and governance, in which “partnerships” — sometimes called “alliances” or “networks” — are dynamic relationships between diverse actors, often representing different organizations, who share mutually agreed objectives and work together to achieve a common goal.^17,23,24^ Measurable characteristics of partnerships can be contrasted with other governance or organizational structures such as contractual or direct implementation models,^16,25^ which have been the historic norm in global health and still constitute the vast majority of decision-making and implementation processes (see Table 1). Unlike contractual relationships where roles and responsibilities are demarcated and enforceable and where goals are often set by one party and communicated vertically to another, partnerships are defined by flexible and dynamic allocation of roles and responsibilities and mutual decision-making and goal-setting. Partnerships are expected to achieve better results, more efficiently, with greater legitimacy or country ownership.^16^

**Table 1:** Partnership vs. Contractual Relationship^a^

	**Partnership Relationship**	**Contractual Relationship**
Characteristics of relationship	Key words: trust, horizontal, mutual, shared	Key words: hierarchical, vertical
Who sets goals/objectives	Mutual, shared and agreed goal among partners	Contracting organization sets goals/objectives
Decision-making	Mutual decision-making process and/or potential to influence decisions	Contracting organization makes decision
Accountability	Reciprocal accountability on outcomes	Unilateral accountability on outputs by contracted to contractor
Organizational identity	The assignment of roles and responsibilities clearly reflects competitive advantage of member organizations	The assignment reflects contracting organization’s interests and purpose in engaging other member organizations

^a^Reference 16.

We reviewed the literature on partnership evaluation to identify and assess existing frameworks or approaches to measuring partnership. Based on this review, we selected a conceptual framework described by Brinkerhoff^16^ – one of the only frameworks describing the holistic set of possible factors from an evaluation perspective and how they inter-relate to lead to added value. Further, this framework has been applied to multiple cases of development partnerships.^26-29^

**Figure 1:**
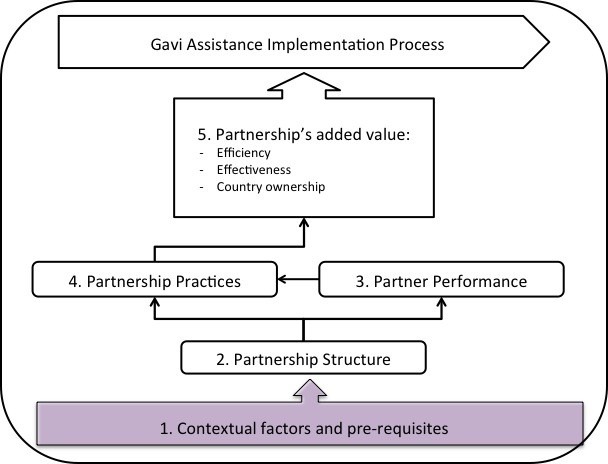
Partnership Analysis Framework.

The framework suggests five dimensions to evaluate, proposing causal relationships between them: context and partnership prerequisites, partnership structure, partnership process, and outcomes, or the added value of the partnership (see Figure 1). The context in which the partnership functions is determined by contextual factors and pre-requisites [1], which may include characteristics of the issue or process, the existence of champions, a history of partnerships and power-sharing mechanisms, and/or the broader political environment. Partnership structure [2] is the overall composition of the partnership, the nature of connections between members, and their respective functions. Partnership process is a function of partner performance [3] and partnership practice [4]. Partner performance describes each partner’s comparative advantage, roles and responsibilities, and their effectiveness in fulfilling these roles. Partnership practice [4], are behaviors and mechanisms among member organizations within a partnership that enhance or diminish the value of a partnership on the process.^16^ Partnership practice in turn contributes to outcomes, or the added value of the partnership. We chose to define and measure added value in line with the FCE’s broader evaluation aims and the focus on effectiveness, efficiency and country ownership [5],^15,30^ and measured perceptions of these outcomes using a survey tool proposed to assess and strengthen partnerships using network analysis.^31^ The effectiveness of the partnership is determined based on the completeness, quality, relevance, and responsiveness of the process. Efficiency is based on the timeliness, responsiveness, costs-savings, and nimbleness of the partnership. We adapted the concept of ‘legitimacy’ to be more specific to Gavi’s core principle of ‘country ownership.’^32^ Country ownership is determined based on the level of autonomy, participation, transparency and satisfaction, adherence to good governance principles, and the legitimacy and sustainability of the partnership. Previous applications of the Partnership Framework have drawn on qualitative or archival data to develop case descriptions.^29^ We expanded this approach to include social network data and triangulation across all data sources.

### Measuring and Describing Partnership Structure

A second gap in the existing empirical research on partnership pertains to the limited use of appropriate tools and analytic methods to measure and describe structure. Structure, which lies at the heart of what differentiates a partnership model from other organizational, governance or business models, is described in the existing partnership literature as “flat,” “horizontal,” or “decentralized” (see again Table 1).^16,20^ We argue that the utility of the partnership framework, and particularly its ability to measure and understand partnership structure, could be improved through the systematic application of SNA concepts and tools.

Social network theory, broadly, suggests that the behaviors and decisions of nodes — individuals, organizations, or any other unit — are determined by those nodes’ relationships with other nodes, as well as the larger structure created by the entirety of the relationships.^33^ In policy and organizational sciences, the structure of individuals or organizations in decision-making, management, planning, and implementation have been correlated with outcomes such as innovation, evidence exchange and use, efficiency, knowledge transfer, and learning.^18-21,34-36^ Structure, in this sense, is empirically measurable and frequently represented by metrics such as density (ie, the proportion of possible ties that exist in the network), centralization (ie, the proportion of ties that go to one focal actor), and diversity (ie, the proportion of ties that are outside- versus within-group).^33^ The systematic measurement of these metrics and their linkage through the framework to the Alliance objectives is a methodological innovation which will hopefully support efforts to evaluate the Alliance partnership model in order to enable it to achieve its intended outcomes moving forward.

## Methods

In this section, we elaborate on the evaluation design and implementation so that it can be easily replicated. We provide sample data collection tools in Supplementary Files 1 and 2.

### Design

Applying the partnership framework required a mixed-methods case study design^37^ embedded in an ongoing prospective evaluation of Alliance support (the FCE).^15^ Among the four FCE countries, Uganda was chosen as the initial case country to implement the partnership analysis because of local interest and the recent completion of a potential case – the development of the funding application to the Alliance for HPV vaccine introduction at the national level. In the partnership analysis approach, the ‘case’ can be defined as a process with a specified outcome of the process, for example an application for new vaccine support, resulting in a submission to the Alliance; or the planning process culminating in the launch of a new vaccine. Defining the case as process facilitates cross-country comparisons and encourages empirical identification of the network of actors who have participated. It ensures that the outcomes (Figure 1, Box 5) are well-defined and measurable, thus, enabling attribution.

The HPV vaccine application process was chosen as a suitable case because of its timeliness in relation to planned data collection and the potential of applying lessons learned to both the ongoing implementation of HPV vaccine in Uganda as well as to other new vaccine applications in Uganda and elsewhere. In addition, it was of theoretic and programmatic interest to understand how a partnership around HPV vaccine, which targets adolescent girls to prevent cervical cancer, might involve a different group of stakeholders than traditional childhood vaccines. As the Partners’ Engagement Framework moves to include ‘expanded’ partners,^14^ the process and outcomes of adding new partners is of particular relevance. Finally, although certain immunization activities (ie, vaccine implementation) involved partners spanning administrative levels and jurisdictions, a national level process was chosen for ease of access to potential respondents and data collection.

### Data Collection

Data collection included document review and in-depth interviews, including a structured network survey, with key informants. The document review component informed the development of topic guides and supported the identification of initial interview respondents. Documents included the final report of Uganda’s HPV vaccine demonstration project,^38^ minutes of application meetings, the application submission to Gavi,^39^ and Gavi’s decision letter.

In-depth semi-structured interviews were conducted with individuals involved in the HPV vaccine application process. Respondents were identified based on the local research team’s in-depth knowledge of the process, and augmented by document review. Interviewers, who were members of the local FCE research teams, followed topic guides based on the partnership framework domains (see Supplementary File 1 for sample topic guide). The aim of the interview was to elicit the respondent’s perception of the overall application process, as well as of constructs within each domain in order to test the relationships between the constructs and domains. All interviews were conducted in Kampala, the capital city of Uganda. Interviews lasted on average 45 minutes. Notes were taken during the interview and were expanded immediately following the interview.

During the interviews, the research team also administered a structured network survey adapted from Provan et al^31^ (see Supplementary File 2 for sample survey). This survey was administered orally at the start of the interview. One attempt to administer it over email was unsuccessful and required visiting that respondent in person; attempts to leave it to the end of the interview resulted in rushing through it, which is a particular challenge when interviewing policy-makers and other policy elites. The survey began by asking the respondent to provide the names of the individual people he or she worked with on the HPV vaccine application. This open-ended ‘name generator’ encourages the empirical identification of the true actors in a network, as opposed to a roster approach (ie, a list of names), which might bias the network towards who is named in formal documentation.^33^ We chose to define our units of survey observation, as well as the node level units of analysis in the networks, as individuals rather than organizations. This decision was based on the local research team’s in-depth understanding of partner dynamics and previous policy network mapping studies that suggested that decision-making was largely relational on an individual level, and that personal behaviors and attributes should not be ignored.^35^

For each name provided, the respondent was also asked whether they shared information with that person during the HPV vaccine application process, how many years they have known that person, and their level of professional trust for that person. Professional trust was defined using the following prompt: “When we say ‘trust,’ we mean can you trust that organization to keep their word, to do a good job, and to respond to your organization’s needs?” and rated on a scale of 1-4 (little trust – high trust; see survey Supplementary File 2 for more details). The question of trust often led to open-ended responses which were recorded and probed on. Interviewers used a fluid approach where they probed on responses during the survey to discuss related domains in the interview topic guide. Following the network survey, respondents were asked to indicate their perceptions of the effectiveness, efficiency, and country ownership of the partnership. To do this, the interviewers read a list of statements adapted from Provan and Milward^18^ and were asked to indicate “occurred” or “did not occur” for each. For example, “Planned activities were executed with greater quality” was one indicator of effectiveness; “Reduction in financial cost of process” was one indicator of efficiency, and “Increased legitimacy of decisions made” was one indicator of country ownership. Negative statements (eg, “Unnecessary management burden on my organization”) were also included (see Supplementary File 2 for complete list of questions). Interviewers found that this component of the survey was easier to complete if they passed the survey instrument to the respondent to read and self-administer. Again, interviewers recorded verbal comments and open-ended responses and probed where appropriate. The survey also included questions about the respondent’s basic job and demographic characteristics, which were completed by the research team prior to the interview to the extent possible. Job and demographic characteristics were also completed, to the extent possible by searching meeting minutes or the Internet, for identified network members who were not surveyed.

### Sampling Procedure and Boundary Definition

A ‘snowball,’ or respondent-driven sampling, approach was used to identify respondents for this study.^33^ This approach was used because it encourages the empirical identification of true actors in a network as opposed to providing a list of people to the respondents which might bisas the network. Due to the prospective nature of the evaluation, the team identified the initial respondents through document review and participant observation during planning meetings. The goal of this process was to identify a sub-set of individuals who were likely to be central to the network without being overly homogenous or closed as a group.

Using an open name generator, names of individuals mentioned in the interviews were added to a master list of network members and these were then approached for an interview. In this approach to defining a network — as opposed to the roster approach which assumes a fixed and known member list — the researcher must define a boundary for data collection but attempt to capture the entire census of network members within that boundary. We chose to stop including new names (ie, the network boundary) when a round of names elicited fewer new names than the previous round. This is a common decision point for dispersed networks, such as policy networks, as it suitably limits bias while not overtaxing resources.^40^

### Data Analysis

The research team read the qualitative interview notes together and coded text segments by hand according to a pre-determined coding structure based on the framework categories. Additional codes included codes for interactions between categories, and the application process for inactivated poliovirus vaccine (IPV) which had occurred more recently than the HPV vaccine application process and was mentioned often during interviews. The team read the coded text segments and wrote a memo for each theme summarizing key interpretations and findings.

Immediately following interviews, network data were entered in matrix form in a MS Excel workbook. Names were entered in rows and columns and the existence of a working relationship was entered in each node-alter cell, weighted by the reported trust score. A second matrix recorded information exchange ties, entered as binary (0 = no tie was reported; 1 = a tie was reported). Respondent attributes and perceived outcomes were entered in a third worksheet, and descriptive analyses performed in Stata.

The two resulting network sociomatrices were imported into UCINET software^41^ where the maximum value of two ties was taken in cases of asymmetry. Density, centralization, and degree centrality were computed based on existing algorithms; a dichotomized version of the working relationship network was also analyzed. Network maps were produced for the networks using NetDraw.^42^

### The Case of Applying to Gavi for the Human Papillomavirus Vaccine Support

Uganda National Expanded Program on Immunization (UNEPI) is responsible for immunization under the Ministry of Health (MoH) in Uganda and is headed by the Assistant Commissioner of Health Services also known as the Expanded Program on Immunization manager (EPI manager). UNEPI is situated in the Department of National Communicable Diseases Control (NDC) within the Directorate of Clinical and Community Services. The UNEPI program is responsible for policy, standards and priority setting, capacity building, coordinating with other stakeholders and partners, resource mobilization, monitoring, and technical support supervision to districts.^43^ UNEPI links with other MoH departments and divisions through Technical Working Groups as well as Senior and Top Management committees. EPI activities are organized during monthly technical committee meetings consisting of EPI country partners led by the EPI manager.

EPI has various partners including (1) Public partners such as National Medical Stores (NMS), district administrations and health facilities; (2) Development partners such as United Nations Children’s Fund (UNICEF), the WHO, and bilateral donors; (3) International non-governmental organizations (INGOs) such as PATH, SABIN Vaccine Institute, African Field Epidemiology Network (AFENET), and Maternal and Child Health Integrated Program (MCHIP).

Uganda has benefited from Alliance support since 2001 with the introduction of hepatitis B vaccine and immunization services support (ISS); since that time it has introduced *Haemophilus influenzae* (Hib) vaccine and pneumococcal conjugate vaccine (PCV), and utilized cash support for injection safety (INS) and HSS windows, receiving a total of $190.6M in Alliance funds to date. Gavi support to Uganda was suspended in 2006 following financial irregularities and was re-commenced in 2012 when those irregularities were resolved.

In Uganda, cervical cancer accounts for 40% of all cancers recorded by the cancer registry, and over 80% of women with cervical cancer are diagnosed with advanced disease.^44^ Cervical cancer is caused by HPV which is a sexually transmitted infection. In Uganda, the annual age-standardized incidence of cervical cancer is estimated at 44.4 per 100 000 women per year and age-standardized mortality rate estimated at 27.2 per 100 000 women per year (2012 estimates).^45^ From WHO projections, deaths are predicted to increase by nearly 80% by 2030 in mostly low- and middle-income countries, Uganda inclusive.^44^ Fortunately, the disease can easily be prevented through HPV vaccinations, screening and treatment. A vaccine to prevent cervical cancer was approved and licensed in the United States in 2004 and in Uganda in 2007.

In order to benefit from Alliance support for HPV vaccine, countries are required to demonstrate their ability to deliver HPV vaccines to adolescent girls prior to application for national rollout. Uganda was among the countries selected by PATH to undertake an *HPV Vaccines project* along with India, Peru, and Vietnam. The project in Uganda was implemented by UNEPI of the MoH with technical support from PATH, and operations research was conducted by the Child Health and Development Centre (CHDC) and PATH. The demonstration project aimed at assessing the feasibility, acceptability and cost of delivering HPV vaccine. The demonstration project was initially implemented in two selected districts in 2008, each testing a different approach, but later scaled up to 12 additional districts in 2009. In Nakasongola district, delivery of HPV vaccine was tested through the biannual Child Days Plus (CDP) approach and the target population was girls 10 years of age. In Ibanda district, a school-based approach was used and the target population was based on school grade (Primary 5) or 10 years of age for girls who were not attending school.

The demonstration was considered successful and the accompanying report indicated that the HPV vaccine was highly acceptable in communities and that implementation was feasible.^38^ A coverage survey in 2009 showed 88.9% coverage with the school-based delivery strategy and 60.7% coverage with the CDP delivery strategy.^38^ Based on the success in the two districts, the demonstration project shifted to using a combined approach of integrating the CDP with school-based immunization, and HPV vaccine immunization was extended to 12 additional districts in 2012. The new combined approach targeted all girls in Primary 4, regardless of age, and 10-year-old girls who were not in school. Vaccination of the first cohort of girls in the 12 new districts began in September 2012, the second dose was administered in November 2012, and the third dose between March and August 2013.

Its success provided evidence to the Government of Uganda about when and how best to introduce the HPV vaccine country wide prior to application for national rollout as this was a Gavi requirement. The HPV vaccine delivery model decided upon then however, has changed due to its feasibility and financial sustainability. Following the successful demonstration, Uganda then made a decision to apply to Gavi for national introduction of HPV vaccine. The application for national introduction of HPV vaccine was prepared between May and September 2013. The initial application was submitted by the Government of Uganda in September 2013 and was approved by Gavi in March 2014.

## Results

### Summary of Descriptive Statistics

The team conducted key informant interviews (KIIs) with seven individuals involved in the HPV vaccine application process between August and October 2014. An additional four network surveys were administered without an in-depth interview, totaling 11 surveys. Respondents were all based at the national level and represented the MoH and partner organizations (see Table 2).

**Table 2:** Organizational Affiliations of Identified Actors

**Organization Type**	**Number**	**%**
MoH	15	38
(CSO/NGO)	8	20
Multilateral	7	18
Government (Not MoH)	2	5
Other	3	8
Gavi	0	0
Research	1	3
Unknown	3	8
Total	39	100

Abbreviations: MoH, Ministry of Health; CSO, civil society organization; NGO, non-governmental organization.

Through the snowball sampling approach we identified a total of 39 individuals who participated in the HPV vaccine application process. Actors in this network had an average of 2.8 ties and reported moderate-high levels of trust for each other (Table 3). The low density score is likely explained by incomplete data collection.

**Table 3:** Network Statistics

**Metric**	**Value** ^a^
Nodes identified	39
Ties	112
Density	0.07
Centralization	0.40
Average degree (ties)	2.8
Average tie weight (ie, reported trust)	3.14

^a^These values are based on analysis of 11 completed network surveys, and thus, care must be taken in interpreting these values alone. Triangulation with other data sources provides a more reliable picture.

Table 4 shows respondents’ mean level of agreement with each of the potential benefits statements. Eighty-four percent of 11 respondents agreed with statements that linked the partnership with increased country ownership, while 79% considered it to have improved effectiveness, and 63% thought it improved efficiency. When assessing potential drawbacks of the partnership, 25% of respondents agreed with the statement that the partnership led to an “unnecessary management burden on my organization” (see Table 4).

**Table 4:** Perceived Benefits of Partnership (n = 11 Respondents Surveyed)

**Benefits**	**% Of Respondents Who Agreed**
Effectiveness	
Planned activities are executed with greater quality	100
Better able to identify the need for, and to acquire additional support	90
Better able to respond to existing challenges, or those that arise during the process	90
Better able to execute introduction activities	78
Increases sustainability of immunization program	35
Mean (effectiveness)	79
Efficiency	
More timely execution of planned activities	80
Leverages each organization’s comparative advantages	70
Reduction in financial cost of process	60
Better allocation of each organizations financial resources	40
Mean (efficiency)	63
Country ownership	
Increases country ownership	90
Increases legitimacy of decisions made	90
Increases fairness of decisions made	89
Increases transparency among partners	80
Increases accountability among partners	70
Mean (country ownership)	84
**Drawbacks**	
Effectiveness	
Creates competition and conflict among member organizations	0
Strained relations within my organization	0
Mean (effectiveness drawbacks)	0
Efficiency	
Unnecessary management burden on my organization	38
Loss of control/autonomy over decisions	11
Forces us to make decisions in a way which is not natural/typical for our organization	11
Mean (efficiency drawbacks)	20
Country ownership	
Not enough credit given to my organization	25
Total (country ownership drawbacks)	25

### Interpreting the Data Through the Partnership Framework

We found that the partnership framework was useful in drawing out relationships between variables. We note three key findings relating the HPV vaccine application partnership to observed outcomes. First, trust was identified in qualitative and quantitative findings as being an important contributor to the perceived effectiveness of the process. Second, network mapping identified the absence of the ministries of education and finance during the application meetings, which may explain observed delays in the planning process after the HPV vaccine grant had been received. Third, respondents perceived the partnership to be only moderately efficient, which might be explained by the lack of clear guidelines or terms of reference around roles and responsibilities. The sections that follow describe these major findings, as well as other findings, according to each domain of the partnership framework.

### Context

Contextual factors such as critical events, existing relationships among potential partners, or politics, can facilitate or block the ability for a partnership to form and function effectively. When describing the HPV vaccine application partnership’s underlying context and history, respondents consistently mentioned past immunization partnership experiences, including that of the HPV demonstration project, the existence of champions, political priority around cervical cancer, and high levels of trust.

Respondents noted that the partnership observed through this study started to form in 2013 with the application and introduction of PCV – the first engagement with Gavi since 2005. A new team of UNEPI managers were appointed in 2013, which respondents identified as having a positive influence in functioning of the partnership and on the timely submission of the HPV vaccine application.

*“Originally, WHO and UNICEF were the major players but in recent years many more partners have come on board. The new EPI management has rejuvenated the partnership and all the partners come together under the EPI technical committee”* (Development partner, 01).

This nascent partnership further evolved and strengthened through the HPV demonstration project, which resulted in positive working relationships between many of the stakeholders.

Another contextual factor that strengthened the partnership was having a “champion” organization. As mentioned earlier, Uganda was one of the countries chosen by PATH to undertake the *HPV vaccine project* and as a result, respondents identified PATH as championing the application process and working behind the scenes to move the process along:

*“PATH played a critical role. PATH continued reminding MoH of the need to prioritize the HPV [vaccine] introduction”* (Development partner, 01).

Qualitative data confirmed the survey finding related to high levels of trust in the network. Respondents typically reported trusting others they worked with, particularly if they had a history of working together. Trust was not always synonymous with perceptions of professional competence and low trust scores are clustered around technical assistance providers who were new to the Ugandan context. The trusting environment was also associated in interviews with country ownership, which was in contrast to the rushed process and lack of ownership in the subsequent IPV application process.

*“The HPV partnership functioned in an environment of trust as mentioned in the survey. There was accountability, honesty and shared goals. If this trust did not exist, then the application process would not be successful”* (Civil society organization, 02).

*“Yes, the HPV application partnership facilitated country ownership of the process, especially when compared to IPV [application process]”* (MoH, 03).

Buy-in and ownership was also attributed to the high levels of political will and priority surrounding HPV vaccine and cervical cancer. The First Lady of Uganda was a champion for HPV vaccine,^46,47^ and the issue (cervical cancer) was considered by respondents a reason for high partner involvement.

### Network Structure Informed Performance and Practices

Our analysis of the network data, confirmed by data from interviews and observation, indicates that the network structure displayed structural attributes consistent with other partnerships: relative decentralization; moderate density in the network core; and high average levels of trust (see Table 3).^20,21,48^ However, network mapping also uncovered ‘missing’ actors.

**Figure 2:**
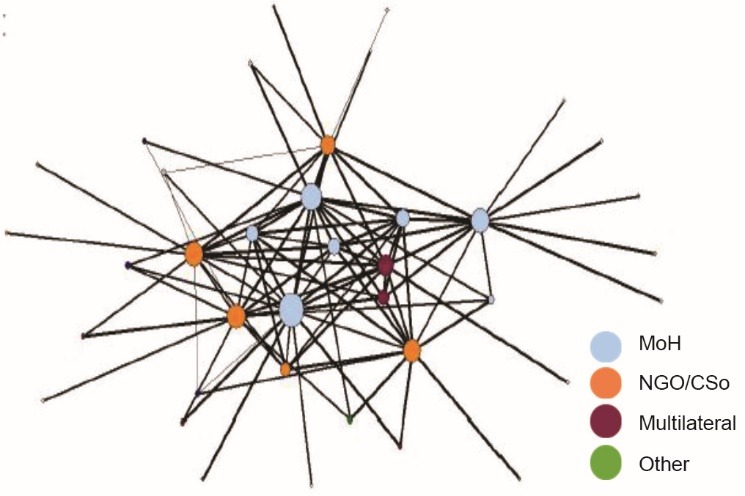
HPV Vaccine Application Partnership Network, Working Together Ties. Ties weighted by reported trust score, nodes sized by degree centrality and colored by organization type. Abbreviations: MoH, Ministry of Health; CSO, civil society organization; NGO, nongovernmental organization.

The network centralization score (0.40) indicates that there was no single leader in this network; although there is some tendency towards UNEPI, followed by WHO and UNICEF. This is illustrated in Figure 2 and is consistent with interview data attributing formal leadership to the MoH, but practical leadership support to other partners, including an advocacy role by PATH:

*“The MoH took a lead in HPV application process… The government is currently taking the leading role in these processes unlike the past. However, PATH played a critical role. PATH continued reminding MoH of the need to prioritize the HPV [vaccine] introduction”* (Development partner).

The network survey helped to identify who was involved, as well as who was not. Given that HPV vaccine is targeted to adolescent girls, and that UNEPI proposed to deliver the vaccine through school-based channels, the involvement of the Ministry of Education (MoE) should have been a necessary condition for application. Some respondents noted that the MoE did not attend application meetings, although their signature was on the application submitted to Gavi:

*“I didn’t see MoE during the application process. They participated in demonstration project but not the application process. We could have actually involved them much more but we just didn’t”* (MoH, 05).

The absence of the MoE was an issue also raised by the Gavi Independent Review Committee (IRC) in their initial review of the application, but the Alliance Board ultimately approved the application. The absence of the MoE was also raised to the EPI program by the FCE team in Uganda, who acted to include the MoE in planning meetings once the application had been approved. As Uganda ultimately decided to deliver HPV vaccine through schools, their participation in the planning process was necessary. This study may have been one contributing factor to their involvement during planning, thus, mitigating potential consequences of their absence.

The Gavi Senior Country Manager was not named. While on one hand their strong engagement is considered essential for Gavi’s success, it is also true that they are meant to provide support for effective implementation of, and not applications for, Gavi support. Only one individual from the Ministry of Finance (MoF) was named, once. The lack of participation from the finance ministry is consistent with other network-based studies of new vaccine decision-making; a finding that is not always apparent from reviewing application documents alone.^49^ Participation of finance ministries in decision-making is recommended as a means of ensuring financial sustainability. As Gavi-eligible countries begin to take on a larger share of vaccine co-financing and transition from Gavi support, there are concerns over the long-term financial sustainability of immunization programs.^50,51^

### Performance and Practices Facilitated Positive Outcomes

So far we have described a partnership that is based on trust and mutual understanding and structured to allow for shared decision-making across a wide range of actors. Yet supportive context and structure are not sufficient to lead to positive outcomes; how the partnership is managed in practice, and whether individuals fulfill their responsibilities and expectations will determine its ultimate effectiveness, efficiency, and country ownership.

Numerous respondents noted that the HPV vaccine application partnership was not formalized in writing; no terms of reference existed to outline composition, roles, and responsibilities. Interviewers probed further on specific mechanisms intended to manage and coordinate immunization partnerships – the Gavi Business Plan and the Inter-agency Coordinating Committee (ICC) – but found they had not played their intended roles. The Gavi Business Plan outlined Alliance partners’ roles and responsibilities for Gavi activities in each country but few respondents were aware of its existence, and thus, which partners were assigned to which specific activities supporting HPV vaccine introduction. The ICC is a national policy advisory committee for immunization that is meant to coordinate partner roles and endorse all Gavi-related decisions, but it was implicated as a final decision-taking body as opposed to one coordinating work processes, roles, or responsibilities across partners.

Respondents differed on the consequences of this for the process; some spoke favorably of the way in which roles were assigned on an as-needed basis. Others found that the process would have been improved if roles were assigned at the beginning. As a result, some respondents noted that some partners were more involved than others.

The consequences of the management issues are perhaps best reflected by whether respondents considered the partnership to have benefitted effectiveness, efficiency, or country ownership. As noted above, the statements that were met with the highest level of agreement were on average related to country ownership and effectiveness, and then efficiency (see Table 4).

While the country ownership outcome scored high overall, only 35% of respondents thought the partnership improved the overall sustainability of the immunization program. A highly functioning HPV vaccine application partnership should add value not only to the HPV vaccine application process, but also to Uganda’s broader EPI program.

## Discussion

As expected, an immunization partnership existed around the HPV vaccine application process for Gavi support, and the partnership was perceived positively in terms of perceived country ownership and effectiveness but slightly less so for efficiency. This study suggests three key drivers of partnership added value: trust; a diverse and inclusive network; and a clear governance mandate.

A key finding was the importance of trust in facilitating the effective functioning of this partnership. Trust existed because of underlying relationships among the network actors, built in part by previous new vaccine introductions and through the HPV vaccine demonstration project. Trust matters; as the Alliance moves forward with the implementation of new forms of country-targeted technical assistance through the Partners’ Engagement Framework, they should remain sensitive to the need to build trust between and within all Alliance partners, including governments.

Our second major finding is related to inclusiveness. On one hand, the absence of the education and finance ministries had the potential to lead to inefficiencies during implementation as their specific knowledge was and is necessary for effective, efficient, and sustainable implementation of the HPV vaccine. In part due to this study and the broader FCE, the MoH ensured the participation of the MoE during the vaccine introduction planning process, ensuring a smooth roll-out of a school-based delivery program. The under-representation of finance ministry staff in vaccine policy decision-making is a persistent issue which must be systematically addressed if long-term programmatic and financial sustainability is to be achieved.

On the other hand, the network was otherwise diverse and inclusive. The composition of the network extended far beyond the Alliance’s ‘core partners’ and demonstrates the utility of empirically mapping such networks. Consistent with partnership principles and network theory, diverse actors ultimately improve the innovativeness and resilience of a network through their ability to access new ideas and resources. In this case, an NGO partner was instrumental in maintaining momentum during the process. However, despite the relative diversity of this network, we note that the network sub-core consists of relatively few individuals, and that the departure of even one could cause major delays or inefficiencies.

Our third driver of partnership added value is clear governance and management structure and processes. Compared to the other outcomes, respondents did not find the partnership efficient. Eighty-four percent of respondents perceived the partnership increased country ownership, compared to 79% considering it to have improved effectiveness, and 63% thought it improved efficiency. The principles that define partnerships, namely shared goals, and decentralized, diverse actors, simultaneously create governance and management challenges. This is particularly true in mandated, versus emergent, networks,^20^ such as the Alliance and most other global health partnerships.

Perceived efficiency and overall governance of the partnership could be improved while retaining its legitimacy. Specifically, this partnership did not report many standardized or formalized procedures or management mechanisms. While the relative lack of formalized rules and procedures enabled flexibility on one hand, we recommend that national immunization partnerships be strengthened to provide a clear governance and management structure and to clarify and coordinate partner roles and responsibilities. Reaffirming the central leadership roles of the EPI program — and strengthening their capacity to govern and manage — will ensure that good governance occurs while also strengthening country ownership.

### Reflections on the Partnership Framework

This case study was driven by the partnership analytic framework.^18^ We see tremendous added value of applying a theoretical framework to inform study design, data collection, and data analysis and to contribute to theory-driven health policy and systems research.^52,53^ The partnership framework domains (context, structure, partner practices, partnership performance, and outcomes) were highly sensitive and specific to the data we collected, and this framework could be applicable to a variety of cases unrelated to Gavi support. We built on the framework’s intended qualitative data collection approach with the use of social network mapping in order to empirically measure who truly worked in the partnership, and the measurable structure and strength of their relationships. Without the network survey, we might not have realized the extent to which the ministries of education and finance were absent from the process, which has had mid-range consequences on the ongoing HPV vaccine planning process. Conversely, the network survey identified key non-traditional Gavi partners who were not named on the application, but yet played an important role in Uganda’s process. This finding may help Uganda’s EPI program as it considers partnership needs and ideal partners for ongoing and future immunization activities.

Our findings also highlight the benefits of the prospective evaluation approach to identify downstream consequences of partnership processes. However, and particularly in an ongoing, prospective evaluation where respondent burden and fatigue is of particular concern, the data-heavy needs of SNA are difficult to achieve. We recommend ongoing experimentation with secondary sources of network data, including meeting attendance, to reduce respondent burden while simultaneously leveraging the power of network science.

The new Partners’ Engagement Framework will potentially improve the transparency and accountability of partners and increase country ownership by involving countries in the identification of their technical assistance needs from partners. However, this study showed that the existence of many capable partners does not ensure clear expectations and management of activities and processes. Ongoing evaluations should track the impact of PEF on partnership networks and their outcomes.

## Strengths and Limitations

Our choice of the HPV vaccine application case was for pragmatic and theoretical reasons. The fact that HPV vaccine is likely to require new or different partners as compared to childhood vaccines may limit the external generalizability of our findings. On the other hand, this same particularity makes it a very relevant case study to inform the implementation and evaluation of the Partners’ Engagement Framework, which will likely bring many new partners into established partnerships. This study was cross-sectional, which means that the network data do not represent the partnership dynamics that certainly exist.

Only 11 network surveys were administered, in part to mitigate respondent burden within the context of a 4-year prospective evaluation. Network data collection is notoriously difficult due to the need to survey the census of the network, but not impossible with adequate time commitment and a supportive context.^36^ This study’s low response rate limits the internal and external validity of the reported SNA measures; however, we have taken great care to present quantitative network findings which were supported by data from qualitative interviews and participant observation. While our data likely provide a more accurate representation of the structure of the network core, we caution that the periphery structure, particularly its density, will be underestimated based on missing ties between non-interviewed nodes. While we measured respondents’ *perceptions* of the partnership’s effectiveness, efficiency, and country ownership during the study, the prospective nature of the Gavi Full Country Evaluation enabled the inclusion of evidence on the mid-term outcomes of the HPV application process and the national implementation of HPV vaccine.

## Conclusion

Despite the particularities of an HPV vaccine partnership, these data and findings can serve as a baseline from which to compare the effects of the new Partners’ Engagement Framework and whether that policy change increases effectiveness, efficiency and country ownership. This study offers a number of important lessons for the design and implementation of the Partners’ Engagement Framework; notably that the quality of relationships matters when considering new partners, and that without clear roles, responsibilities, and terms of references, adding new partners is only likely to decrease efficiency further. Further efforts to test the partnership framework should ultimately lead to clear recommendations for governments and partners outlining how to best structure and manage partnerships to achieve critical global health goals.

The partnership framework and approach presented here can be applied to measure the health and performance of other global health partnerships to ensure that they are meeting the complex challenges they are designed to address. As partnerships become an increasingly common approach to health and development, it is important to ensure they are performing optimally and intervene when they are not. The partnership framework combines SNA and partnership theory into a single useful and adaptable framework that evaluators or policy-makers can apply to understand and intervene to strengthen networks, to ultimately improve global health more effectively, efficiently, and legitimately.

## Acknowledgments

We would like to acknowledge the support and assistance of other team members on this project: Faith Namugaya and Moses Kamya from IDRC, Kampala, Uganda; Julie Rajaratnam, Ashwin Budden, and Jeff Bernson from PATH, Seattle, WA, USA; and Steve Lim from the Institute of Health Metrics and Evaluation at the University of Washington, Seattle, WA, USA. Most of all we are grateful to the study participants in Uganda and particularly the Ugandan MoH for the time and knowledge they gave to this study. This work was supported by Gavi, the Vaccine Alliance as part of the Gavi FCE. The funder played no role in the work including design and conduct of the study, data collection, data management, data analysis and interpretation, preparation, review, and approval of the manuscript.

## Ethical issues

Ethical approval for the Gavi FCE, which includes this study, was secured from the University of Washington Institutional Review Board and the Makerere University School of Biomedical Sciences Higher Degrees Research and Ethics Committee.

## Competing interests

Authors declare that they have no competing interests.

## Authors’ contributions

JS, NS, PW, DH, and JB were involved in the conception and design of the study; CK, GA, JS were involved in the acquisition of data; CK, PW, GA, JS, and EC analysed the data; CK, JS, EC, NS, and PW drafted the manuscript with critical revisions from JS, DH, and JB.

## Authors’ affiliations

^1^Infectious Diseases Research Collaboration (IDRC), Kampala, Uganda. ^2^PATH Seattle, WA, USA. ^3^Makerere University School of Public Health, Kampala, Uganda. ^4^Department of Public Health Sciences, Karolinska Institutet, Stockholm, Sweden. ^5^The INDEPTH Network, Maternal, Newborn and Child Health Working Group, Accra, Ghana. ^6^George Washington University, Washington, DC, USA. ^7^Management Sciences for Health, Arlington, VA, USA.

## Supplementary Files

Supplementary Files 1

Supplementary Files 2 Supplementary Files 1 and 2 contain sample data collection tools.

## Key messages

**Implications for policy makers**

- Policy-makers can use the partnership framework and network mapping to understand the actors in the partnerships that affect their work.
- Based on an understanding of the relevant stakeholders, policy-makers, and those who support them can work to ensure that all relevant government and non-government stakeholders are represented in the process.
- Policy-makers and those who support them can strengthen partnerships and processes by ensuring sufficient coordination mechanisms, including the existence of terms of reference or a partnership coordinator. As partnerships grow they require additional investment in coordination.

**Implications for public**

The public, and particularly the health of the public, is shaped by policies that are developed and implemented by networks of actors, where the ability to work together to develop or implement a government policy depends on who is involved and how they are connected. This study shows that the structure and quality of connections between people involved in immunization policy in Uganda affected their ability to work together effectively, efficiently, and with a sense of legitimacy. By improving the evaluation of partnerships using the partnership framework and approach, this research can help policy-makers make better policies by ensuring that the right people are involved, and better policies will ultimately improve the quality of life for all citizens.
