# Supplementary material for: Evaluating Global Health Partnerships: A Case Study of a Gavi HPV Vaccine Application Process in Uganda
Source: Int J Health Policy Manag. 2016 Oct 26;6(6):327–38. doi: 10.15171/ijhpm.2016.137 (PMC5458794; doi:10.15171/ijhpm.2016.137)
Supplement: Supplementary Files 1 [file ijhpm-6-327-s001.pdf]

IJHPM
International Journal of Health Policy and Management

Supplementary File 1. In-depth interview guide

Key Informant Interview (KII) guide

Topic: Partnership analysis for new vaccine support application processes in Uganda: A case study of HPV

Key words to Listen for:

When respondent uses the below words in their response, please pay close attention to how the words were used, the context in which they are used, and try to capture the direct quotation as accurately as possible.

Context and pre-requisites

Questions	
I would like to start by asking you about working together, generally. In your own words, what does "partnership" mean in the context of GAVI support and immunization programmes? Do you think a partnership exists in your country in relation to immunizations, generally?	Probe: How is the partnership different from other approaches to implementing donor assistance, or improving health programmes?<br>Probe: What is the goal of this partnership?<br>Probe: Is there one partnership, or many partnerships at the national level. Sub-nationally? Regionally or globally?<br>Probe: Did partnership exist for the HPV application process? How did it differ from the general immunization partnership(s)? How did it differ from the partnership engaged in the recent IPV application process?
Can you tell me a bit about the history of the partnership for the HPV application?	Probe: Who drove the development of the partnership for this application? (GAVI, UNICEF, WHO, MoH; local vs. regional or global actors); role of PATH demonstration project.<br>Probe: How did various roles or goals changed over the course of the HPV application process?<br>Probe: What lessons were learned from the partnership for the HPV application?
Did champions exist, that facilitated the functioning of the HPV partnership?	Probe: Role of GAVI SCM, inside MoH, other partners
Did the HPV partnership function in an environment of trust? ("Professional trust" means you trust them to do a good job and work with integrity)	Probe: Did partners trust other partners' integrity, honesty, accountability?<br>Probe: Did partners trust other partners' competence, knowledge, shared goals?<br>Probe: Is trust important?<br>Probe: Is the level of trust you have described for this HPV partnership typical of other health/development partnerships or activities? How is it the same? How does it differ?
Are there contextual factors specific to Uganda that facilitated or blocked the HPV partnership(s)' work?	Probe: Existence of previous successful (or failed) partnerships<br>Probe: Level of decentralization; level of coordination between MoH departments<br>Probe: Economic or political events<br>Probe: Role/strategic interest of GAVI in this country<br>Probe: Political priority around HPV<br>Probe: Role of demonstration project in the formation or functioning of the current HPV partnership<br>Probe: Scientific evidence of high burden of Human papilloma virus and Cancer of cervix

1. Partnership structure: How is the partnership structured?

**Questions**	
In the survey, you told me whom you worked with in relation to HPV application process. Can you tell me who else worked on the HPV application process?	Probe: National, sub-national, regional or global actors<br>Probe: How were members identified and invited to work on the HPV application?<br>Probe: Did all stakeholders participate equally?<br>Probe: Did the same people attend all meetings? Was there significant (problematic) turn-over?<br>Probe: Were any stakeholders missing who should have been involved/ more involved?<br>Probe: Do you think any stakeholders had too large a role and should have been involved less?<br>Probe: What role did Ministry of Education (MoE) play in HPV application process? How did they become involved as a partner? When did they become involved?<br>Probe: Role of PATH and Merck from demonstration project
How were stakeholders and their activities coordinated for the HPV application process? Was there a management mechanism?	Probe: Was there a defined mechanism for managing the activities of partners during the HPV application process? Does a TOR exist for these mechanisms? *[Listen for ICC, HSCC, other ad-hoc committees, Coordinating Committee]*<br>Probe: Would these mechanisms exist without GAVI, or did some of them already exist in-country?<br>Probe: What was the role of informal communication for the HPV application process?<br>Probe: How effective were the various management structures/groups at managing the partnership? Provide examples of what worked and what could be improved?<br>Probe: What was the role of global or regional partners in the coordination of the HPV partnership?<br>Probe: Was the coordination and management mechanism of partners for the HPV application different than the coordination and management of the most recent IPV application? If so, how?
Adaptations:<br>*Depending on the form of support or activity being studied, are there different partners involved or management mechanisms?*<br>Ie, Different partners for HPV, different policy positions?<br>*Consider how an "unplanned event" might be traced back to partnership structure. Is it possible that the structure was too centralized? Too unconnected?*	

2. Partner Performance: To what degree does an individual partner fulfill their roles and responsibilities?

Questions	
What were the roles and responsibilities of each partner in the HPV application process?	Probe for each partner mentioned earlier.<br>Probe: Were roles and responsibilities aligned with partners' organizational competencies and comparative advantages?<br>Probe: How were roles and responsibilities determined within the partnership(s)? Were these roles and responsibilities determined by regional or headquarter offices, or were they decided locally?<br>Probe: Are stakeholders aware of the roles and responsibilities outlined in the GAVI business plan pertaining to the HPV application? [Note that the business plan says that WHO is responsible for providing support for HPV applications, and AVI TAC is responsible for providing support to the "HPV country application"]<br>Probe: Was there any confusion about roles and responsibilities for the HPV application preparation? Were partners aware of other partners' roles and responsibilities?<br>Probe: Did duplication in roles/responsibilities ever occur between partners for the HPV application? How has such a scenario been dealt with?
Did partners comply with their agreed roles and responsibilities for the HPV application?	Probe: Did all partners fulfill their roles and responsibilities?<br>Probe: What happened if they didn't?
Can you tell me about a time when an individual or an organization in the partnership went beyond their roles and responsibilities to ensure that the HPV application process was successful?	

3. Partnership Practices: To what degree does the partnership operate as a “partnership”

**Questions**	
Now I would like to discuss how the partnership functions as a whole. Can you provide me with your perspective on how well partners worked together in the process of HPV application?	Probe: Did some partners work better together than others? Why? How did this vary for planning for the application development process, data gathering and sharing, application development (including the plan and budget), obtaining necessary application endorsements, submission to GAVI, response to etc.?<br>Probe: How would you compare the function of the partnership for the HPV application with the IPV application?
How was the partnership held accountable for its work?	Probe: Did partners collect data or meet to discuss the performance of the partnership?<br>Probe: Were partners open to adjusting their performance and practices in order to improve?<br>Probe: Is the partnership accountable in any way to citizens?
How did partners share information relevant to the partnership and its activities?	Probe: Were responses to information timely?<br>Probe: Are partner organizations willing to share information with other partners?
In your own words, please tell me how decisions were made by partners in regards to new vaccine support application, specifically the HPV application?	Probe: Did different partners have different levels of decision-making authority?<br>Probe: Were all partners included?<br>Probe: How was decision-making similar or different during the IPV application process?
In your own words, please tell me how resources were allocated within the partnership?	Probe: Who provided resources (financial, technical, human resources, network, expertise), to whom?<br>Probe: Flexibility in meeting changing needs?<br>Probe: Did partners perceive resource allocation decisions to be fair?

4. Partnership added value

**Questions**	
Overall, what effect do you think the partnership(s) have had on the **efficiency** of applying for HPV GAVI support and strengthening the immunization programme?	Probe: Has the partnership made it easier to move through the process in a timely way?<br>Probe: Has the partnership somehow delayed progress?
Overall, what effect do you think the partnership(s) have had on the **effectiveness** of the application process for HPV?	Probe: Has this partnership either facilitated or blocked effective implementation, as measured by outputs (ie, timely submission of application) and outcomes (ie, funds secured in time)?
Overall, what effect do you think the partnership(s) have had on the **legitimacy** of the application process for HPV?	Probe: Does the partnership facilitate or block country ownership of HPV application process?
