# Supplementary material for: Evaluating Global Health Partnerships: A Case Study of a Gavi HPV Vaccine Application Process in Uganda
Source: Int J Health Policy Manag. 2016 Oct 26;6(6):327–38. doi: 10.15171/ijhpm.2016.137 (PMC5458794; doi:10.15171/ijhpm.2016.137)
Supplement: Supplementary Files 2 — Supplementary Files 1 and 2 contain sample data collection tools. [file ijhpm-6-327-s002.pdf]

IJHPM
International Journal of Health Policy and Management

Supplementary File 2. Network survey

Partner Survey

Background Information [to be completed by interviewer and confirmed during interview where necessary]

1. Activity/event being studied: HPV application process______________________
2. Respondent's name: ___________________________
3. Respondent's organization: __________________________
4. Respondent's job title: __________________________
5. Location (city) of respondent's organization: ___________________________________
6. Is respondent's organization an international, national, or sub-national organization? ______________
7. Number of years respondent has been at his/her present organization: ___________
8. Interview Date: ________________
9. Name of Interviewer: ____________________________
10. Name of Note taker: _____________________________

Script to interviewer

Today's interview focuses on the national immunization programme, and particularly the application process for HPV vaccination that occurred leading up to its submission in September 2013.

You have been identified as being involved in the HPV application. We would like to know who you worked together with in the context of the HPV application process in order to understand who was involved, what the various roles and responsibilities were, and to better understand the process overall. The results of this survey and interview can be used to inform future immunization activities, processes and partnerships.

[Begin survey]

Can you tell me the names of other immunization stakeholders that you worked together with during the process to prepare the HPV application? Which organization do they work for?

[Allow respondent to list all names, giving them time and silence to think of additional names. Do not mention or probe on specific people's names; let the respondent list names inductively.]

Thank you. For each name, I am going to ask you a set of questions for them. First, you named [insert name].

For [first name on form], did you exchange information with them to inform the HPV application? Information can include reports, studies, training plans, budgets, work-related advice, etc. [record response as check in the box]

[Repeat question for each name on form]

For [first name on form], for how many years have you known this person, either personally or professionally? [record response as number in the box]

Now we would like to ask you a question about [insert person's name] organization. We would like you to reflect on the overall level of trust **you have for that organization**. Can you describe your trust for that organization? [take notes]

[If needed]: When we say 'trust,' we mean can you trust that organization to keep their word, to do a good job, and to respond to your organization's needs?

Now if you were to categorize what you just told me, would you rate your trust for their organization as: 1 = poor relationship (little trust); 2 = fair relationship (some trust); 3 = good relationship (trust); 4 = excellent relationship (high trust).

[circle appropriate number on survey form]

[Repeat question for each name on form]

Go to next page for partner survey

**Individuals the respondent works with**	**I exchanged information with them**	**Duration of relationship**	**Professional trust**
(Write name)	Check if yes	Write number of years	(Please circle)
Name:<br>Org:			1 2 3 4
Name:<br>Org:			1 2 3 4
Name:<br>Org:			1 2 3 4
Name:<br>Org:			1 2 3 4
Name:<br>Org:			1 2 3 4
Name:<br>Org:			1 2 3 4
Name:<br>Org:			1 2 3 4
Name:<br>Org:			1 2 3 4
Name:<br>Org:			1 2 3 4

We would now like to know what the benefits and drawbacks were from working together with other individuals and organizations in the context of the HPV application. For each possible benefit or drawback listed, please indicate, by placing a check in the appropriate box, whether you think that because of working together with other individuals and organizations, the benefit/drawback occurred or not. Check (☐) only one box for each benefit/drawback.

Benefits:	Occurred	Did not occur
a. Better able to execute activities	☐	☐
b. More timely execution of planned activities	☐	☐
c. Planned activities were executed with greater quality	☐	☐
d. Better able to identify the need for, and to acquire additional support	☐	☐
e. Better able to respond to existing challenges, or those that arose during the process	☐	☐
f. Better allocation of each organization’s financial resources	☐	☐
g. Reduction in financial cost of process	☐	☐
h. Leveraged each organizations’ comparative advantages	☐	☐
i. Increased sustainability of immunization programme	☐	☐
j. Increased country ownership	☐	☐
k. Increased transparency among partners	☐	☐
l. Increased accountability among partners	☐	☐
m. Increased legitimacy of decisions made	☐	☐
n. Increased fairness of decisions made	☐	☐
o. Other benefits:		

Drawback:	Occurred	Did not occur
a. Unnecessary management burden on my organization	☐	☐
b. Created competition and conflict among member organizations	☐	☐
c. Loss of control/autonomy over decisions	☐	☐
d. Strained relations within my organization	☐	☐
e. Not enough credit given to my organization	☐	☐
f. Forced us to make decisions in a way which was not natural/typical for our organization	☐	☐
g. Other drawbacks:	☐	☐
